# Afterthoughts about bovine spongiform encephalopathy and variant Creutzfeldt-Jakob disease.

**DOI:** 10.3201/eid0707.010744

**Published:** 2001

**Authors:** P Brown

**Affiliations:** National Institutes of Health, Bethesda, MD, USA.


### Afterthoughts about Bovine Spongiform Encephalopathy and Variant Creutzfeldt-Jakob Disease

The recent review (1) of bovine spongiform encephalopathy (BSE) and variant Creutzfeldt-Jakob disease (vCJD) stimulated a large number of questions and comments about additional issues discussed in this commentary.

### Advice for Travelers to Europe

The most frequently asked question concerned travel to Europe this summer: what is safe to eat, and what should be avoided. In principle, beef and beef products should no longer carry any risk because the European Community, as well as individual European countries, have taken measures to prevent the entry of potentially contaminated products into the human food chain. These measures include regulations to end the recycling of mammalian protein into ruminant feed (extended by some countries to feed used for all mammalian species); removal of the heads and vertebral columns of slaughtered cattle before further processing; and prohibition of certain nervous system and visceral tissues for human consumption. In the United Kingdom, mechanically removed meat, even after removal of the vertebral column, is prohibited for human use.

Nevertheless, because the extent of BSE outbreaks in most European countries is still not known—more and more cases are being discovered through active surveillance using sensitive immunologic tests—and because there is always an imprecise lead time before the implementation of precautionary measures, it would probably be wise to remain wary of beef products in continental European countries (and certain other countries) in which BSE already exists or is likely to appear, and in which safeguards may not yet be fully implemented. The European Commission of Food Safety has published a global country-by-country BSE risk analysis, with continuing web site updates as new information becomes available at http://europa.eu.int/comm/food/fs/sc/ssc/outcome_en.html.

It is also important to reemphasize that beef as such is not dangerous; the villains were almost certainly beef products that (in the past) contained mechanically recovered meat contaminated by nervous system tissue. These beef products were often added to such precooked items as sausages, hot dogs, bologna and other luncheon meats, canned beef products, beef stews and broths, and meat pies—in short, any product containing beef in a precooked and often unrecognizable form. Cooking cannot be guaranteed to sterilize BSE infectivity: experiments using different strains of spongiform encephalopathy agents have shown only partial inactivation at temperatures as high as 350°C (662°F) (2,3).

### Dairy and Other Bovine-Derived Products

Many readers asked about the safety of milk and dairy products. Persuasive evidence indicates that these important consumables are risk free: milk from infected cows has been fed to, and injected into the brain of, susceptible RIII mice without transmitting disease. Even more convincingly, calves suckling gallons of milk from their infected mothers have not contracted the disease (4).

Questions have also arisen about the safety of other products derived from bovine sources, such as gelatin and tallow derivatives, which find their way into a dazzling array of items eaten or used by humans. These include products as varied as jellybeans, cosmetics, and vaccines. Scientific advisory committees in many countries, including the United States, have addressed these questions and have concluded that infectivity in the tissues used as sources of these products is either low or nonexistent, and that even if existent, infectivity would in most cases be destroyed by the processes used in product manufacturing. Nevertheless, depending on estimates of risk inherent in tissue sources and use of different products, industry has been either obliged or strongly urged to ensure BSE-free status of source cattle and to include processing steps known to destroy infectivity.

### Nonbovine Products

Products derived from nonbovine species, including sheep, pigs, and chickens (both meat and eggs) should also be entirely safe, as there is no evidence that any of these species is naturally susceptible to BSE. Scrapie-infected sheep tissues have been eaten by humans for centuries without causing disease (5), and although pigs can be experimentally infected with BSE by direct brain inoculation, attempts to induce infection by feeding have not succeeded (6). Chickens are resistant to both natural and experimental infection.

### The American Scene

Concerns have been raised about BSE status in the United States and about the safety of foods and other products from domestic bovine sources. BSE could occur in the United States only as a result of 1) cases arising de novo in domestic cattle, 2) cases arising from exposure to spongiform encephalopathies in other domestic species, or 3) cases arising from the importation of infected cattle or livestock feed. With respect to de novo cases, it is possible that cattle (and perhaps all mammals) are subject to the same rate of spontaneously occurring disease as that of CJD in humans, approximately one case per million animals per year. If true, this incidence has so far escaped detection, despite extensive recent search for cases.

One oft-repeated but misguided objection to this statement is that very few apparently healthy cattle have been examined. However, if we wish to know whether or not a given disease exists in a population, we do not concentrate on asymptomatic persons, but instead on population subsets at highest risk. For example, if we wished to know whether or not cases of Alzheimer's disease occurred in a given country, we would first examine older adults, not the general population. The same principle applies to BSE. Although asymptomatic cattle with BSE might be infected, it is far more probable that BSE will be diagnosed in animals with neurologic signs or at least some indication of illness (even if atypical), and it is precisely these animals that are being tested.

Microscopy examination of brain specimens from >12,000 cattle categorized as downers (abnormal recumbency from any cause) or with suspected neurologic disease has failed to reveal a single case of BSE. Brain specimens from >3,000 of these cattle have also been tested immunologically for prion protein. By way of comparison, in Switzerland (a country in which BSE has been carefully researched), tests for the protein have been carried out in 40,000 animals, including 15,000 downer cattle, and yielded approximately one diseased cow for every 1,000 examined, half of these from the downer group. The comparison is statistically significant; that is, testing already completed in the United States would have detected BSE if it existed at the incidence level of Switzerland. Testing of cattle in the United States will continue, and many thousands more animals will be examined in the next few years, which will provide increasing assurance of BSE-free status.

With respect to cattle or cattle feed imported from the United Kingdom (or other countries in which BSE has occurred), the United States led the way in taking measures to prevent or correct any such occurrences. During the years before the importation of live ruminants and ruminant products was banned (in 1989), 500 cattle and a single shipment of 12 tons of meat and bone meal feed were imported from the United Kingdom. Almost all of the cattle were traced, and if still living, were slaughtered and destroyed. We can say today that any animal or animal feed that might have been contaminated did not transmit the infection because with an incubation period of approximately 5 years, BSE would already have spread through recycled carcass cattle feed to cause a recognizable outbreak of disease.

Import barriers have since been extended to include all countries in which BSE exists or which have not convincingly demonstrated its nonexistence. Thus, the argument that undetected cases of BSE might be present in the United States, although impossible to disprove, is not supported by current evidence. Because of the everpresent risk for human error, vigilance is still required to see that all established preventive measures are properly and continuously monitored.

### Allied Diseases

Three varieties of spongiform encephalopathy present in the United States, scrapie in sheep, transmissible mink encephalopathy (TME), and chronic wasting disease (CWD) of deer and elk, under the right circumstances may be capable of infecting other animal species. Scrapie first appeared in the United States in 1947 in Michigan sheep of British origin that had been imported from Canada and has since spread to most regions of the USA. Scrapie has not been convincingly shown to cause disease in any other species (apart from goats), despite its certain inclusion in rendering mixes for livestock until the 1997 mammalian to ruminant feed ban. Exactly why species barriers have not been crossed is unclear but may be due to a relatively low flock incidence of scrapie and a relatively small proportion of sheep to cattle in rendering plants, such that the very low amount of infectivity entering the total rendering mix does not survive processing into livestock feed.

TME was also first reported in 1947 on a Wisconsin mink ranch and has occurred in several further outbreaks on mink ranches both in the United States and abroad. TME was originally thought to have resulted from feeding (scrapie-infected) sheep carcasses to the mink, but in one U.S. outbreak in 1985, epidemiologic study indicated that the dietary source might have been downer cattle carcasses (7). No experimental verification of this hypothesis was undertaken, no recurrence of TME has been identified in the United States during the past decade, and no outbreak of TME has occurred in any country (including the United Kingdom) that has BSE in native-born animals.

CWD was first recognized in 1967 in captive deer on a Colorado wildlife research facility. It occurs endemically in wild deer in contiguous sections of northcentral Colorado and southeastern Wyoming and episodically on elk farms along the eastern border states of the Rocky Mountains. No disease in humans or other animals has been attributed to CWD, but the potential for disease is very real: infected tissues could be eaten by predators or enjoyed by aficionados of wild game, and carcasses could be rendered for feed that (by error) could find its way to cattle. Regional hunters and elk farmers have been alerted to the risks, but more attention at the national level is urgently needed.

### What if…?

What would result from the mistaken feeding of contaminated mammalian protein to a herd of cattle? During the 1980s, millions of cows in the United Kingdom were eating at least half pound of meat and bone meal dietary supplement each day, some of which was certainly contaminated, yet the incidence of BSE in affected herds never exceeded 2% to 3%. Today in the United States, the worst that could happen after a contaminated feed incident would be that a few cattle might be infected and come to slaughter unrecognized, but without a sequence of similar errors, no infectious tissue would be recycled in the livestock food chain, and a regulatory breakdown of this magnitude is virtually impossible.

Nevertheless, if even a single case of BSE were to be discovered in the United States, the economic and perceived public health consequences could be immense. How would the current package of preventive measures stand up to future judgment? With generally high marks, although a few points of vulnerability still exist (the elimination of which would substantially dislocate segments of the rendering and livestock industries):

- The 1997 ban on using mammalian protein in ruminant feed exempts plate waste from restaurants (which could contain bovine brain or paraspinal ganglia in the uneaten remains of some cuts of meat). They could be recycled to cattle in feed produced by rendering plants.
- Feed for ruminants and nonruminants can be processed in the same feed mills, creating the potential for cross-contamination. Rendered carcasses of deer and elk with chronic wasting disease could conceivably in this way be fed to cattle.
- If farmers or ranchers mistakenly or deliberately use nonruminant feed for ruminants, the mammalian to ruminant feed ban would be bypassed. Spontaneous TSE occurring in nonmammalian species, if it occurs, would also escape the mammalian to ruminant feed ban.
- Unlike the European Union, the United States does not mandate that the rendering process be capable of sterilizing BSE infectivity. (Most feed mills render carcasses at 134°C without the concomitant use of steam and pressure, and thus cannot be guaranteed to sterilize).

- Mechanically separated meat expressed from crushed carcasses can be added to cooked and uncooked meat products, up to a concentration of 30% by weight. Since 1997, spinal cord has been removed, but the vertebral column (including the paraspinal ganglia) can still be processed and used in many products: hot dogs, sausages, canned beef, luncheon meats, and soups and stews. A recently introduced and much safer process (advanced meat recovery) has not yet completely replaced the crushing method.
- Organs known to be infectious in cattle with BSE (including brain) are not prohibited from human consumption.
- Glandular dietary supplements containing various animal organ powders, including cattle brain, were often imported from the United Kingdom or countries in continental Europe until the U.S. Department of Agriculture import ban in 1989. The ban relies on proper labeling of the shipment and can be abused.

As an alternative to the entire issue of precautions against the occurrence and spread of BSE, we must finally ask, what would be the economic consequences of eliminating animal protein from livestock feed or replacing it with plant protein?

The rendering industry would disappear, and the incineration industry would expand in conjunction with the production of nutrition crops such as soybeans. Would plant protein be as effective as animal protein? Is the public prepared to pay more for meat or eat only as much as can be produced from range-fed animals? These are not trivial questions, and the answers will need to be weighed against the overarching issue of public health.

**Paul Brown**
Senior Investigator, National Institutes of Health,
Bethesda, Maryland, USA

### References

1. Brown P, Will RG, Bradley R, Asher DM, Detwiler L. Bovine spongiform encephalopathy and variant Creutzfeldt-Jakob disease: background, evolution, and current concerns. Emerg Infect Dis 2001;7:6-16.
2. Brown P, Rau EH, Johnson BK, Bacote AE, Gibbs CJ Jr, Gajdusek DC. New studies on the heat resistance of hamster-adapted scrapie agent: threshold survival after ashing at 600°C suggests an inorganic template of replication. Proc Nat Acad Sci U S A 2000;97:3418-21.
3. Steele PJ, Taylor DM, Fernie K, McConnell I. Persistance of TSE infectivity: dry heat treatments. Meeting abstracts: Characterization and diagnosis of prion diseases in animals and man. Tubingen, 23-25 Sep 1999; p. 154.
4. Wilesmith JW, Ryan JBM. Absence of BSE in the offspring of pedigree suckler cows affected by BSE in Great Britain. Vet Rec 1997;141:250-1.
5. Brown P, Bradley R. 1755 and all that: a historical primer of transmissible spongiform encephalopathy. BMJ 1998;317:1688-92.
6. Dawson M, Wells GAH, Parker BNJ, Scott AC. Primary parenteral transmission of bovine spongiform encephalopathy to the pig. Vet Rec 1990;127:338.
7. Marsh RF, Bessen RA, Lehmann S, Hartsough GR. Epidemiological and experimental studies on a new incident of transmissible mink encephalopathy. J Gen Virol 1991;72:589-94.
